# Vimentin Intermediate Filaments: A Paradigm Shift From Static Structure to Dynamic Cytoplasmic Network

**DOI:** 10.1002/bies.70125

**Published:** 2026-03-15

**Authors:** Bhuvanasundar Renganathan, Stephen A. Adam, Vladimir I. Gelfand

**Affiliations:** ^1^ Department of Cell and Developmental Biology Feinberg School of Medicine Northwestern University Chicago USA

**Keywords:** cytoskeletal crosstalk, filament dynamics and organelle positioning, intracellular transport, Vimentin intermediate filaments

## Abstract

Recent advances in live‐cell imaging, super‐resolution microscopy, labeling techniques and cryo‐electron microscopy reveal vimentin intermediate filaments (VIFs) as adaptable polymers that couple mechanical stability with rapid remodeling. In this review, we highlight recent findings and discuss how VIFs function as dynamic, interpenetrating networks with actin microfilaments and microtubules, coordinating cytoskeletal architecture while simultaneously facilitating organelle positioning and influencing cellular behavior. We also propose a hybrid transport model to capture the diverse modes of VIF cellular interactions. This emerging framework positions VIFs as dynamic integrators of cytoskeletal organization and intracellular logistics, with broad implications for understanding cell mechanics, migration, and disease.

## Graphical abstract

Vimentin intermediate filaments form a dynamic, motile network that interacts with microfilaments and microtubules. We propose a hybrid transport model to explain vimentin filament dynamics through cytoskeletal crosstalk and organelle‐associated transport. Thus, intermediate filaments are an active coordinator of cell mechanics, migration, and intracellular organization.

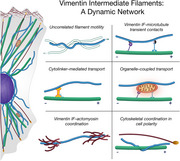

## 1 Introduction: Vimentin Intermediate Filaments

The cytoskeleton of eukaryotic cells consists of three principal filament systems: microtubules (MTs), intermediate filaments (IFs), and actin microfilaments (F‐actin) [1, 2]. The roles of MTs and F‐actin in intracellular transport [3], cell polarity [4, 5], and motility [6, 7] have been extensively characterized. Although IFs have been actively investigated in these processes, their behavior at the level of individual filaments has been more challenging to resolve. Among the various IF subtypes, vimentin is the most widely expressed IF protein in mesenchymal and migratory cells [8, 9, 10]. Historically, the role of VIFs was primarily viewed as that of structural elements, assembling into mechanically resilient, apolar networks to protect cells from mechanical stress and maintain cellular integrity.

Although IFs, and VIFs in particular, have long been recognized as mechanically robust networks [11], their dynamic organization, modes of transport, and coordination with other cytoskeletal systems have remained less clearly defined than those of MTs and F‐actin. This gap reflects both historical conceptual frameworks and technical challenges in visualizing individual filament‐level behaviors within dense cytoskeletal networks. Recent advances in live‐cell imaging, super‐resolution microscopy, and molecular labeling strategies now provide the means to directly interrogate VIF dynamics with substantially improved spatial and temporal resolution, motivating a re‐evaluation of longstanding models of IF organization.

In this review, we revisit historical views of VIF organization, outline the technological advances that have enabled mechanistic insights into their dynamics, and present an emerging paradigm in which VIFs function not merely as mechanical scaffolds but as responsive and motile filament systems that are central to cytoskeletal coordination and cellular adaptability.

## 2 Historical Assumptions and Early Views

Intermediate filaments emerged as distinct cytoskeletal structures through ultrastructural and biochemical studies in the late 1960s and 1970s [12, 13, 14]. Ishikawa and colleagues first demonstrated the existence of a class of 10 nm cytoplasmic filaments with a diameter intermediate between F‐actin and MTs [12]. Subsequent studies identified multiple IF subtypes in various cell lines [15, 16, 17, 18, 19]. In 1978, Franke et al. introduced the term “vimentin”, derived from the Latin *vimentum*, (flexible rods, lattices, or wickerwork) to describe one type of these filaments [20]. During the 1980s–1990s, electron microscopy studies employing rotary‐shadowing techniques portrayed VIFs as smooth, unbranched cables approximately 10 nm in diameter described as rope‐like structural elements [21, 22, 23, 24].

For decades, the VIF network was primarily viewed as a stable mechanical scaffold—a dense, apolar, detergent‐insoluble meshwork that buffers cells against mechanical stress [25, 26, 27, 28]. Vimentin knockout studies in mice initially appeared to support a view of limited functional significance, as the mice developed and reproduced without overt defects under laboratory conditions [29]. However, subsequent analyses of these mice under stress conditions revealed the roles for vimentin in wound healing and tissue repair. More detailed characterization demonstrated that VIFs actively regulate the organization of actin cables, contractile dynamics, and mechanotransduction [30] —revealing dynamic functions whose complexity explained the minimal knockout phenotype.

Early biochemical and cell biological investigations across multiple intermediate filament (IF) types—including vimentin, keratins, neurofilaments, and glial fibrillary acidic protein (GFAP)—revealed that these polymers are not static entities but exhibit dynamic exchange with a soluble subunit pool [31, 32, 33, 34, 35]. Subsequent studies demonstrated that IFs (such as vimentin IFs and neurofilaments) undergo cycles of severing and reannealing, processes that modulate filament length and organization within the cytoplasm [34, 36, 37, 38]. Together, these data demonstrate that type III IFs are not inert polymers but rather dynamic structures subject to active cellular regulation. Owing to their lack of intrinsic polarity and absence of readily observable behaviors such as treadmilling or dynamic instability, they were often perceived as less dynamic than F‐actin and MTs [39, 40, 41]. Moreover, the limited spatial and temporal resolution of light microscopy precluded direct visualization of filament‐level transport, remodeling, or long‐range redistribution.

## 3 Vimentin Molecular Architecture and Hierarchical Assembly

**FIGURE 1:**
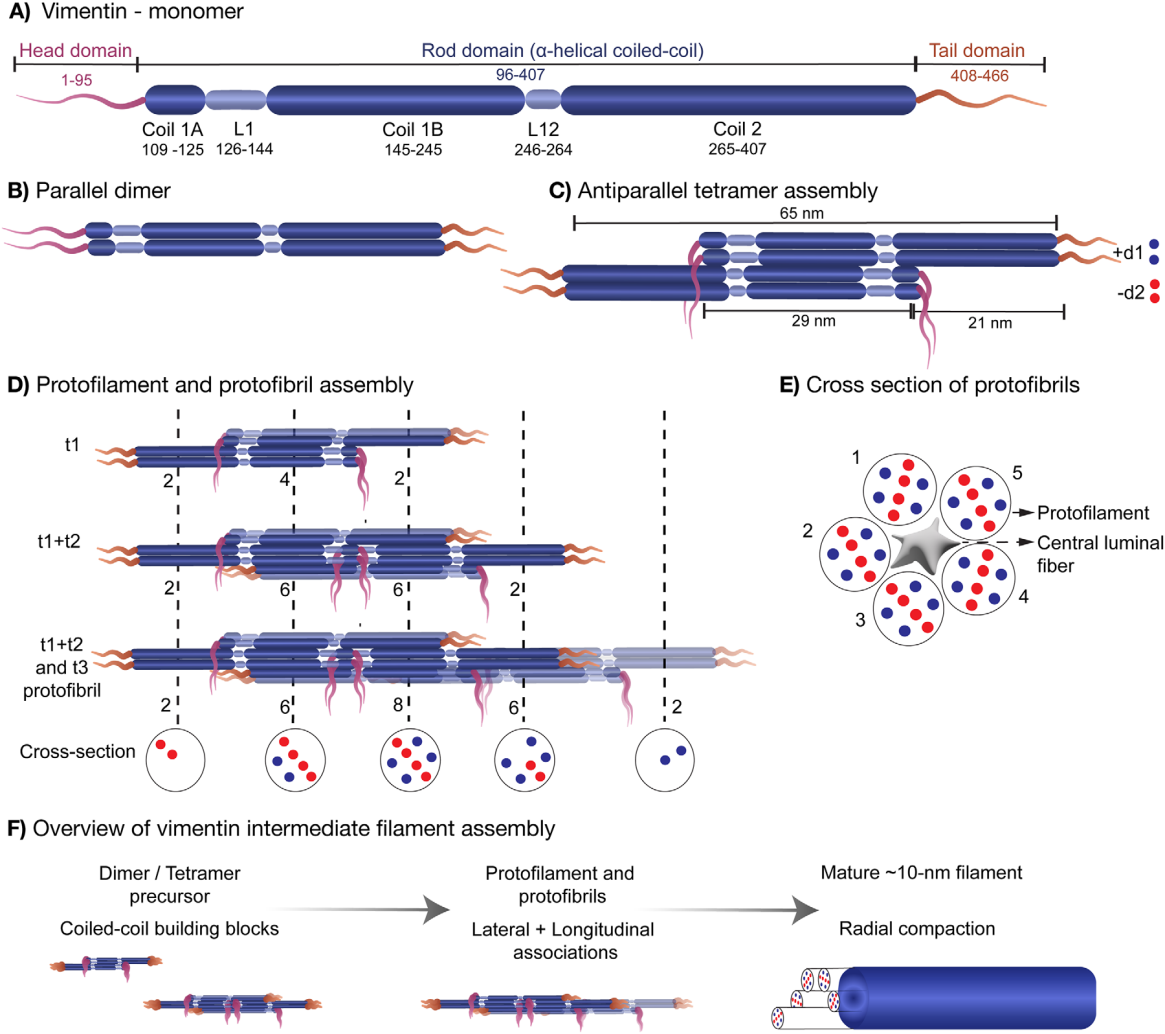
Hierarchical assembly of vimentin intermediate filaments. (A) Vimentin monomer organization. Schematic representation of the vimentin monomer showing its tripartite domain organization: an intrinsically disordered N‐terminal head domain (residues 1–95), a central α‐helical rod domain (residues 96–407), and an intrinsically disordered C‐terminal tail domain (residues 408–466). The rod domain is subdivided into coiled‐coil segments 1A, 1B, and 2, separated by flexible linker regions (L1, and L12). Residue boundaries are indicated. (B) Parallel dimer formation. Two vimentin monomers assemble into a parallel, in‐register coiled‐coil dimer through interactions along the α‐helical rod domain, forming the elementary building block (∼46 nm long). The head and tail domains remain flexible and project outward, preserving molecular polarity within the dimer. (C) Antiparallel Tetramer assembly (A11 configuration). Two parallel dimers associate in an antiparallel, half‐staggered A11 configuration, overlapping at the 1B coiled‐coil regions to form a soluble tetramer (∼65 nm long). Dimers oriented with their N‐termini in the positive orientation are denoted as +d1 (blue), whereas dimers in the opposite orientation are denoted as −d2 (red). This antiparallel association yields a non‐polar, soluble tetramer that serves as the basic unit for higher‐order assembly. Tetramer formation is driven by charge complementarity and hydrophobic interactions along the 1B region. (D) Protofilament and protofibril formation. Tetramers undergo longitudinal annealing to form protofilaments (∼2–4 chains/cross‐section, 2–3 nm diameter), followed by lateral association of 4–8 tetramers to generate protofibrils (∼8 chains/octameric cross‐section, 4–5 nm diameter). Sequential assembly stages (t1: first lateral layer; t1+t2: two layers; t1+t2+t3: protofibril) illustrate progressive compaction toward mature ∼10–12 nm filaments (helical pitch ∼45–60 nm/turn). Dashed vertical lines denote repeating longitudinal units. Numbers (2, 4, 6, 8) indicate the number of 1B coiled‐coil domains in each filament cross‐section. Blue/red circles represent positively/negatively oriented tetramers. (E) Cross‐section of a protofibril. Representative cross‐section of a protofibril composed of five protofilaments arranged around a central luminal amorphous fiber (gray). Individual protofilaments are numbered. Colored dots represent the relative orientation of tetramers within each protofilament, highlighting mixed polarity and internal organization of the protofibril. (F) Overview of vimentin intermediate filament assembly. Protofibrils laterally associate and undergo radial compaction to form mature vimentin intermediate filaments (∼10 nm diameter, helical pitch ∼45–60 nm/turn). Assembly intermediates are shown schematically and are not drawn to scale.

Vimentin, a type III IF protein, is predominantly expressed in mesenchymal cells such as fibroblasts, as well as in cells at certain developmental and transformed states [42, 43]. The vimentin monomer (∼466 amino acids, ∼54 kDa) has a tripartite organization: a central α‐helical rod domain flanked by intrinsically disordered N‐terminal head and C‐terminal tail domains (Figure 1A). The rod domain (∼310 amino acids) is organized into three α‐helical segments (coil 1A, coil 1B, and coil 2) separated by flexible linkers [44, 45, 46, 47]. VIFs assemble through a spectrum of intermediate structures, including small particles or dots, squiggles, unit‐length filaments (ULFs), and short filaments that subsequently anneal and mature into extended networks [48, 49, 50]. The molecular assembly begins with dimerization of the central coiled‐coil domain of vimentin monomers in a parallel, in‐register (∼45–50 nm), followed by antiparallel, half‐staggered association of dimers to form a ∼60 nm non‐polar tetramer (Figure 1B and C) [31, 51, 52]. Tetramers serve as the fundamental building block for polymerization, representing the asymmetric unit of the mature filament helix [26, 53, 54].

Recent cryo‐EM and cryo‐tomography studies have fundamentally revised the structural model of VIF [55]. Rather than forming four‐ or eight‐stranded rope‐like assemblies, VIFs are composed of five protofibrils organized into a tube‐like structure with a slight helical twist. Each protofibril contains three tetramers (eight polypeptide chains) see Figure 1D and E. The filament exhibits helical symmetry with a 42.5 Å axial rise and 73.7° rotational twist, producing a repeat every ∼207 Å—consistent with the ∼21 nm periodicity noted in earlier rotary‐shadowing studies. These modular, five‐protofibril helices provide a structural basis for how VIFs can remain both strong and dynamically reconfigurable [55]. The defined VIF structure is assembled primarily from the A11 tetramer configuration [53]. However, other forms of tetramers (A22, A12, and ACN) have been reported and may contribute to filament polymorphism [52, 56, 57, 58]. The coexistence of multiple possible tetramer configurations suggests a degree of structural heterogeneity that could support continuous filament remodeling. Cryo‐EM analyses have further revealed the role of N‐ and C‐terminal domains in the filament assembly and elongation. These domains contribute directly to filament architecture: the head domains converge to form a central luminal fiber that “glues” protofibrils, while the tail domains interact with adjacent protofibrils (Figure 1E). Such interdigitated arrangements demonstrate how disordered regions complement the structured coiled‐coil core to create a polymer with both strength and flexibility.

VIF assembly is highly responsive to post‐translational modifications (PTMs) [59], particularly phosphorylation [60, 61, 62], ubiquitination, sumoylation [63], and glycosylation. Phosphorylation of serine residues within the head domain promotes filament disassembly during mitosis and cell migration, ensuring rapid turnover and reorganization [64, 65]. Conversely, tail domain phosphorylation and O‐GlcNAc glycosylation [66, 67] modulate lateral protofibril associations, thereby fine‐tuning filament flexibility and interactions with partner proteins.

In filament assembly and maturation, factors such as tetramer concentration and ionic strength play critical roles [68, 69]. It has been proposed that high local concentrations of vimentin tetramers may promote liquid‐liquid phase separation (LLPS), leading to condensate formation that could accelerate ULF formation and subsequent filament elongation by concentrating assembly‐competent tetramers. The intrinsically disordered head domain is a primary driver of this condensate behavior [70, 71], although the precise molecular mechanisms remain under investigation. Nevertheless, droplet size, stability, and composition vary based on vimentin PTMs such as phosphorylation and oxidation, as well as the presence of associated proteins such as motor proteins and cytolinkers [72, 73]. Together, these parameters regulate the formation, lifetime, and dynamics of vimentin assembly precursors, providing an additional layer of control over VIF organization.

Together, these insights redefine VIFs not as inert mechanical cables but as dynamically tunable nanostructures. Their architecture—coiled‐coil core, disordered domain interactions, PTM regulation and mesoscale assembly mechanism such as LLPS—endows them with the capacity to withstand mechanical stress while serving as responsive platforms for cytoskeletal coordination, organelle organization, and signaling.

## 4 Overcoming Technical Barriers: Advances in VIF Network Imaging

A major obstacle in understanding VIF dynamics has been their small diameter, apolar nature, and spatial distribution within cells. VIFs in some cells are densely packed around the nucleus transitioning into sparser arrays toward the cell periphery, rendering the inner network particularly challenging to resolve optically.  In widefield fluorescence microscopy, perinuclear filaments appear as a poorly resolved bright mass due to overlapping structures and out‐of‐focus light, making single‐filament behavior impossible to assess [74, 75]. Although confocal microscopy improves optical sectioning, it is still insufficient for resolving individual filaments in crowded regions [76], thereby biasing early live‐cell imaging and quantitative analyses toward peripheral VIFs, where individual filaments clusters or precursors could be more readily distinguished.

Imaging techniques such as fluorescence recovery after photobleaching (FRAP) [48, 77] and photoconversion [78, 79] suggested that VIFs are dynamic, but the lack of spatial resolution prevented adequate discrimination of individual filament‐level transport. Time‐lapse imaging using total internal reflection fluorescence (TIRF) microscopy enabled visualization of segments of individual filament close to the substrate [80, 81], but this approach was confined to a thin imaging zone (∼100–200 nm), limiting the continuity of filament tracking. As a result, longer‐range filament movements across different regions of the cell could not be characterized, leading to an incomplete understanding of VIF dynamics.

To overcome these limitations, our group developed a single‐particle tracking (SPT) approach based on sparse fiduciary labeling of VIFs using the SunTag labeling system [82]. By labeling only a small fraction of filaments, this approach circumvents signal overlap and crowding, enabling unambiguous visualization of individual filament dynamics throughout the entire cytoplasmic volume, including the densely packed perinuclear region. As with any epitope‐based amplification strategy, multivalent tags such as SunTag could in principle influence VIF organization. In addition, the RPE cells used in these studies express both vimentin and keratin IFs, which form distinct but interpenetrating networks; while keratin–vimentin interactions are not expected to dominate the measured vimentin dynamics, subtle influences on filament behavior cannot be formally excluded. Nonetheless, ultrastructural and dynamic analyses support incorporation of tagged subunits into morphologically normal, dynamically competent vimentin filaments under sparse‐labeling conditions, although subtle network‐level effects cannot be ruled out. Furthermore, SunTag‐labeled VIFs show dynamics comparable to photoconvertible (mEOS)‐labeled VIFs, indicating that SunTag labeling does not noticeably alter VIF behavior.

**FIGURE 2:**
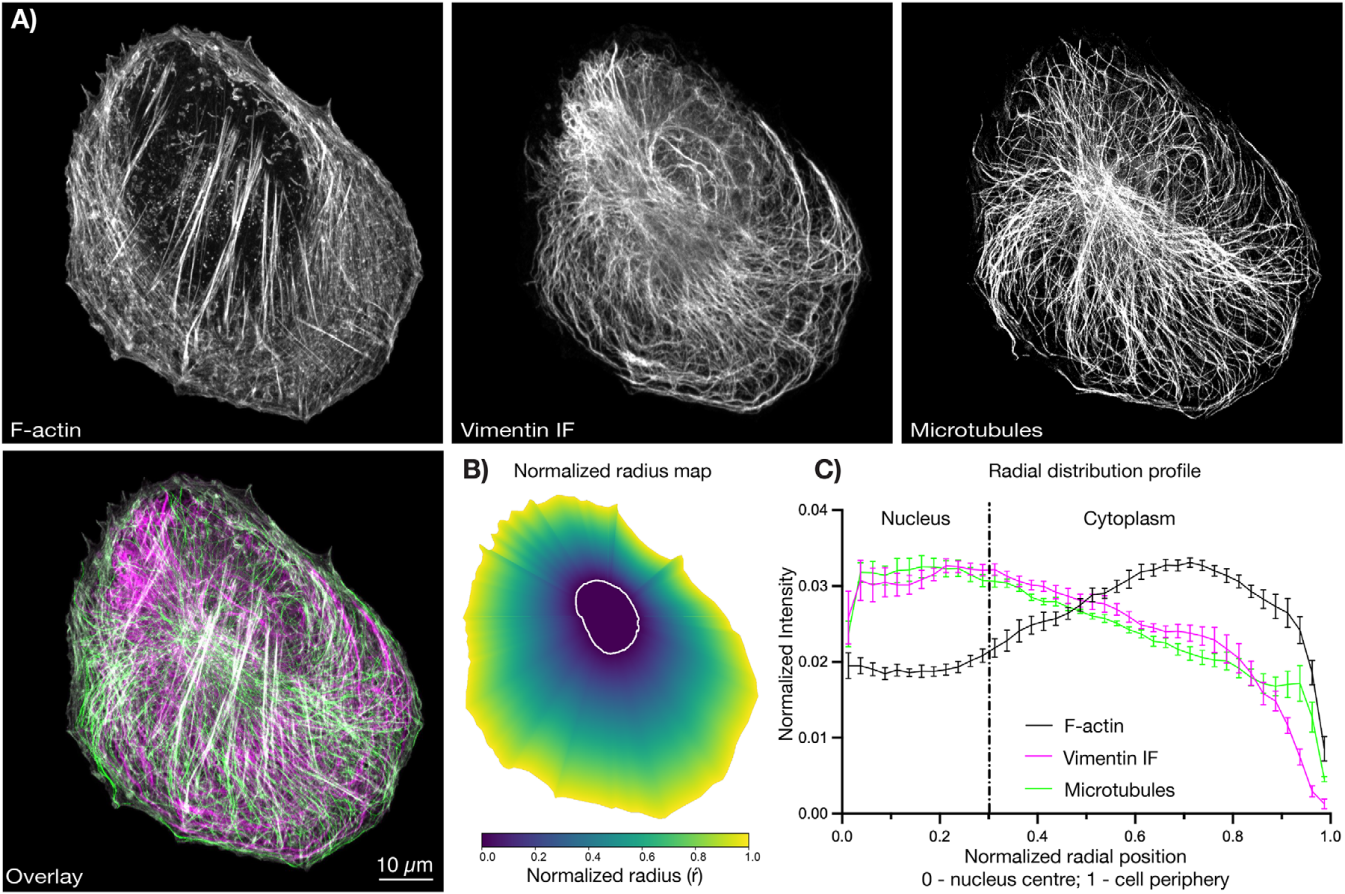
Spatial organization and radial distribution of the three major cytoskeletal networks. (original figure and data). (A) Representative immunofluorescence confocal images of Factin, vimentin intermediate filaments (IF), and microtubules in a retinal pigment epithelial cell, shown individually in grayscale and as a merged color overlay (Factin, white; vimentin IF, magenta; microtubules, and green). Scale bar, 10 µm. (B) Normalized radial position map derived from nuclear and cell outlines for a representative cell. The nuclear boundary (white line) defines radius 0, and the cell periphery defines radius 1. (C) Radial intensity profiles of Factin (black), vimentin IF (magenta), and microtubules (green), plotted as mean normalized intensity ± SEM as a function of normalized radial position (*n* = 7 individual cells). The vertical dashed line marks the average nuclear–cytoplasmic boundary. Factin intensity is biased toward the cell periphery, whereas vimentin IFs and microtubules are enriched in the perinuclear region and at intermediate radii, illustrating the complementary radial organization of the three cytoskeletal systems.

**FIGURE 3:**
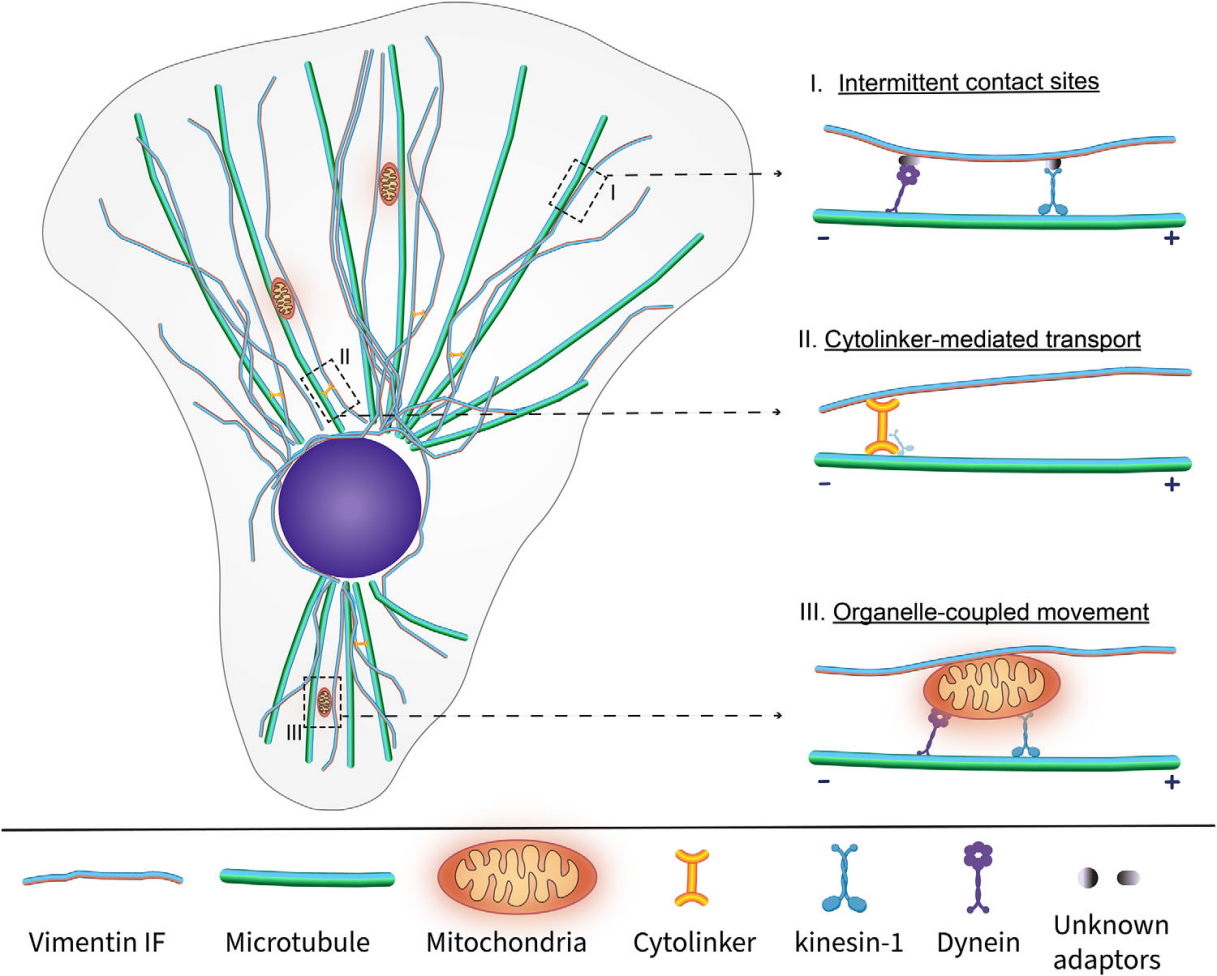
Hybrid transport model for vimentin intermediate filament dynamics. Schematic illustrating a hybrid framework in which VIF repositioning arises from overlapping and complementary transport mechanisms rather than continuous filament translocation. This model integrates intermittent contacts, cytoskeletal transport, and organelle coupling, consistent with observations that VIF exhibit microtubule‐dependent motility involving kinesin‐ and dynein‐ based transport. (I) Intermittent contact sites: Sparse and transient alignments or close contacts between VIFs and microtubules provide localized access for motor‐driven transport without destabilizing the global network. (II) Cytolinker‐mediated transport: Molecular motors may engage VIFs indirectly through cytolinkers such as plectin, enabling selective repositioning of individual filaments while maintaining overall network integrity. (III) Organelle‐coupled movement: VIFs physically associate with membrane‐bound organelles such as mitochondria or endoplasmic reticulum, which themselves undergo microtubule‐based motility, thereby driving co‐transport of attached filament segments. Because organelles maintain defined steady‐state positioning, this mode supports directed motion while constraining net redistribution of the VIF network. Collectively, these mechanisms reconcile strong microtubule dependence with sparse filament–microtubule alignment and provide a conceptual basis for how dynamic VIF mobility is balanced by local retention, anchoring, and network assembly–disassembly to maintain a stable, heterogeneous steady‐state organization. Importantly, these mechanisms are not mutually exclusive and may act sequentially or in parallel depending on cellular context.

This SunTag labeling approach provided direct evidence that VIFs are active throughout the whole cell. VIFs exhibit multiple modes of motility, including persistent directed transport, mixed‐polarity movements, intermittent pauses, and locally confined behavior, in agreement with earlier observations of intermediate filament dynamics [83, 84]. Notably, perinuclear filaments in a dense network exhibit dynamic behavior comparable to that of peripheral filaments. Quantitative analysis revealed that close to ∼10% of VIFs were engaged in directed motion at any given time, with similar fractions observed in both central and peripheral regions. These findings suggest that previously considered spatial differences in VIF dynamics were largely due to imaging problems rather than actual biological compartmentalization.

Correlative 3D electron microscopy using focused ion beam–scanning electron microscopy (FIB‐SEM) further revealed that vimentin “bundles” are not rigid, crosslinked cables like bundles of F‐actin but loosely associated filament assemblies. Our three‐dimensional reconstructions showed variable bundle widths (range: 50–300 nm) and heterogeneous filament packing, with individual filaments frequently emerging to interact with neighboring structures. This loose organization explains how motor proteins can extract single filaments for transport without dismantling entire bundles—a finding consistent with SPT observations of uncorrelated filament movements within clusters.

A more comprehensive picture of VIF organization is emerging through the integration of complementary techniques‐ SIM for super‐resolution network architecture, cryo‐electron tomography for nanoscale structural details, and SunTag‐SPT for filament motility. Recent advances enabling the visualization of individual filament behavior within the cellular milieu have opened new avenues for understanding the roles of VIFs in diverse cellular contexts and mechanistic processes. Continued improvements in imaging methodologies and minimally perturbative labeling strategies (such as the recently reported FilaBuster [85]) are expected to further refine our ability to interrogate intermediate filament dynamics in their native cellular context Table 1 provides a choronological overview of advances in imaging technologies and the key discoveries that shaped our understanding of VIFs biology.

**TABLE 1:** Timeline of imaging techniques impacting VIFs: discoveries and insights.

Timeline	Technique	Discovery	Impact / Key insight	Ref.
1960–70	Transmission Electron microscopy	Provided ultrastructural characterization of IFs at nanometer scale.	Determined filament with 10 nm diameter and distinguished from F‐actin and MTs.	[12]
1970–80	Transmission Electron microscopy	Identification of various classes of IF in different cell types (from late 60s to early 70s)	Established vimentin/keratin/desmin/neurofilament as distinct classes based on cell type	[17, 19]
	Widefield Fluorescence	Enabled first visualization of VIFs/IF networks in cells	Established ubiquity in mesenchymal cells and their dense cytoplasmic distribution	[20, 39, 74, 138]
1990–2000	Confocal live imaging	Introduced dynamic studies of vimentin in living cells. Allowed high‐resolution 3D imaging and time‐lapse of vimentin remodeling.	Demonstrated that IFs undergo rapid turnover and are not static scaffolds. Revealed intricate network reorganization and interactions with actin and MTs	[36, 139]
	Fluorescence Recovery After Photobleaching	Enabled quantitative measurement of vimentin subunit exchange and filament motility.	Revealed continuous subunit turnover, establishing IFs as highly dynamic polymers	[36, 77, 140]
	Atomic force microscopy	Measured nanoscale stiffness of single filaments and networks.	Demonstrated vimentin's role in conferring elasticity and resilience under stress	[141]
2010–20	TIRF imaging	Visualized assembly and disassembly of short vimentin precursors at the plasma membrane	Provided direct live‐cell evidence of precursor dynamics and peripheral remodeling.	[80, 81]
	Photoactivatable/ Photoconvertible labelling	Enabled selective labeling of vimentin filaments	Revealed localized filament renewal and directional transport pathways	[81, 142]
	Super‐resolution (STED, dSTORM, PALM, etc.)	Overcame diffraction limits to resolve sub‐filamentous architecture.	Uncovered axial periodicity, and higher‐order VIF organization	[56, 143]
	Nanobody/Chromobody labeling	Enabled endogenous tracking of vimentin without overexpression.	Allowed physiological measurement of assembly/disassembly in native environments.	[144]
2020–25	Cryo‐EM/Cryo‐ET	Delivered atomic‐level 3D models of vimentin filaments. (2007 and 2024)	Unveiled authentic helical filament architecture and molecular basis of mechanical stability	[55, 145]
	Single particle tracking	Tracked individual vimentin filaments in live cells.	Quantified filament motility, bundle dynamics, and heterogeneity within networks, uncovering semi‐coherent organization of bundles.	[82]
	FIB‐SEM volumetric imaging	Generated near‐isotropic 3D reconstructions of VIF network in whole cells	Mapped network topology and spatial organization in whole cell	[82]
	Multiphoton & multicolor imaging	Enabled deep‐tissue visualization of vimentin.	Connected subcellular filament characteristics to tissue‐level structure and mechanics.	[146]
	Optogenetic manipulation	Mature VIF filaments can be disassembled using Halotag dye	To depolymerize the filament structure locally and globally	[85]

## 5 VIF Interaction With Microtubules

VIFs engage in extensive functional crosstalk with MTs and F‐actin to coordinate intracellular organization and dynamics (Figure 2 illustrates the spatial distribution of the three cytoskeletal systems in a typical cell) [1, 86]. In light‐microscopy studies, VIF motility often appeared tightly coupled to the microtubule network, such that moving VIF structures seemingly follow microtubule tracks over extended distances in two‐dimensional projections [81]. This movement‐associated concordance creates the impression of a scaffold‐based model where MTs serve as a continuous guide for VIF positioning and transport, with VIFs acting as largely passive cargo. This interpretation was further reinforced by pharmacological perturbations showing that microtubule depolymerization led to rapid collapse of the VIF network toward the cell center, consistent with a loss of transport support [87]. Live‐cell imaging and biochemical studies have established that VIF motility depends on MTs and their associated motors, kinesin‐1 and cytoplasmic dynein, with both anterograde and retrograde movements contributing to network organization [88, 89, 90]. Importantly, the movement of VIFs does not depend on microtubule polymerization dynamics. This suggests that VIF motility arises from active, motor‐driven transport rather than from the growth or shrinkage of microtubules [81, 82]. However, light microscopy cannot resolve the three‐dimensional geometry or continuity of VIF–microtubule engagement, leaving open whether effective transport requires long‐range filament co‐alignment or localized interactions— a question recently addressed by 3D‐FIBSEM.

High‐resolution 3D reconstructions have fundamentally transformed this view. Quantitative volumetric analyses reveal that VIFs and MTs occupy largely distinct cytoplasmic territories rather than forming continuous parallel tracks [82]. VIF clusters lie on average ∼309 nm from the nearest microtubule surface, and more than 85% of microtubule‐associated voxels are separated by at least 50 nm from any VIF. Only a minority (∼11%) of individual VIFs extend outward from bundles to make close contact with MTs. Complementary in vitro optical trapping assays from the Köster laboratory provide further evidence for weak direct interactions, with a binding probability of 6.1 × 10^−^^4^ per vimentin unit‐length filament–tubulin dimer pair, independent of filament geometry [91]. Although VIF and MTs are largely separate and rarely aligned over long distances, VIF transport and organization are strictly MT‐dependent, suggesting complex molecular bridging between vimentin and MTs, although the precise nature of such linkages remains unresolved.

Despite their spatial separation, VIF–MT interactions are functionally bidirectional. VIFs actively stabilize and template MTs by reducing catastrophe frequency and enhancing rescue events, thereby extending filament lifetimes both in vitro and in cells [91, 92]. Sparse but strategically placed contacts between VIFs and MTs may serve as anchoring points that reinforce microtubule arrays, particularly under mechanical stress. Beyond stabilization, long‐lived VIF networks can also template microtubule organization by biasing where MTs persist and are maintained, contributing to cell polarity and directional migration [92].  How such limited and discontinuous physical interactions enable robust microtubule stabilization and templating remains an interesting question, underscoring the likely central role of molecular bridging factors in integrating the VIFs and microtubule cytoskeletons. While MTs play a central role in VIF transport, these interactions take place within an integrated tri‐cytoskeletal framework, highlighting the need to examine how vimentin interfaces with the F‐actin network

## 6 VIF Interaction With Actin Microfilaments

Earlier studies showed that VIFs are predominantly localized in the perinuclear cytoplasm, while F‐actin is enriched at the cell cortex, lamellipodia, and focal adhesions [93]. This apparent spatial segregation led to the widely accepted view that the two filament systems operated in parallel, contributing separately to mechanical resilience (VIFs) and dynamic force generation (F‐actin) [94]. This prevailing idea dominated the field for decades, reinforced by conventional immunofluorescence microscopy that revealed distinct subcellular distributions and suggested minimal structural crosstalk between these networks.

However, advanced high‐resolution imaging has challenged this segregation model. Structured illumination microscopy (SIM) and cryo‐electron tomography (cryo‐ET) reveal that VIFs and F‐actin bundles form extensive interpenetrating networks (IPNs) within the cortical cytoplasm rather than occupying segregated domains. VIFs weave through and interconnect adjacent stress fibers, creating composite meshworks that mechanically reinforce F‐actin bundles. Quantitative measurements confirm remarkably close spatial proximity (∼11 nm center‐to‐center distances) between VIFs and F‐actin in stress fiber regions [94]. Beyond structural interpenetration, VIFs and F‐actin work synergistically in the cellular context. Consistent with these observations, cytolinker proteins of the plakin family, particularly plectin, and actin‐bundling proteins such as fimbrin, provide direct molecular linkages between vimentin and F‐actin, offering mechanistic explanations for the observed interpenetrating networks. This structural integration enhances contractile recovery following mechanical stress and supports actomyosin force generation [95], highlighting the dual architectural and regulatory roles of VIFs.

Beyond structural interactions, F‐actin appears to be involved in the vimentin assembly processes. Phase separated vimentin condensates have been observed to associate with actin stress fibers, where they subsequently elongate into filaments, suggesting that F‐actin may facilitate both nucleation and directional elongation of VIFs [96]. Although the molecular interaction mediating this behavior remains largely undefined, these observations raise the possibility that F‐actin can serve as a key component of the local biochemical environment—one among several interacting proteins and cofactors that spatially and temporally tune vimentin assembly and network organization.

Pharmacological depolymerization of F‐actin or inhibition of myosin motors alters VIF motility and organization [78, 97, 98]. VIF association with the actomyosin structure thus likely contributes to VIF positioning and possibly short‐range displacement along F‐actin. While the detailed mechanisms remain under investigation, this F‐actin‐based process complements the long‐range transport of VIF by microtubule‐based motors. This reflects a general transport model in which long‐range microtubule‐based movement is coupled to local actomyosin‐dependent repositioning [99]. Together, this underscores the contribution of F‐actin to VIF transport and organization.

In the context of cell migration, VIFs contribute to actomyosin contractility modulation through matrix stiffness sensing. On soft substrates, VIF enhances force transmission by reinforcing actomyosin networks, whereas on stiff substrates, it preferentially reinforces MTs against compressive loads, thereby tuning cellular mechanosensing [100, 101, 102].  This dual functionality involves sophisticated biochemical signaling cascades: VIFs downregulate RhoA activity by modulating the microtubule‐associated Rho‐guanine nucleotide exchange factor GEF‐H1 [103], with vimentin depletion leading to increased GEF‐H1 phosphorylation, elevated RhoA activity, and enhanced stress fiber assembly [93, 104]. In addition, small GTPases such as RhoA and Rac1 reciprocally regulate VIF organization via downstream kinases, including ROCK and, which modulate vimentin phosphorylation and filament reorganization. These pathways underscore that VIF remodeling is tightly coordinated with actin cytoskeletal signaling.

The integration of MTs into vimentin‐F‐actin crosstalk creates a tri‐cytoskeletal coordination system essential for directed cell movement. VIF simultaneously stabilizes MTs while modulating actomyosin contractility, enabling cells to maintain persistent polarity during migration through complex tissue environments. VIF transport and organization by both microtubule‐based motors (kinesin‐1 and dynein) and myosin‐mediated repositioning ensures that the IF network can dynamically respond to changing mechanical and chemical cues while preserving the structural integrity necessary for effective cell locomotion. This progression from apparent segregation to intimate structural, organizational, and functional integration positions VIFs as key contributors bridging F‐actin and microtubule networks, significantly advancing models of cytoskeletal coordination in mechanosensing and migration.

## 7 VIF and Organelle Positioning: From Cage Structures to Dynamic Platform

The role of VIFs in organelle positioning was first described through the notion of “vimentin cages” [105]. Early electron microscopy and immunofluorescence studies suggested that VIFs formed dense perinuclear networks extending to the cell periphery, compartmentalizing cytoplasmic space and encasing organelles [8]. This reinforced the prevailing view that IFs served mainly as static scaffolds.

However, contemporary evidence from live‐cell imaging, super‐resolution microscopy, and correlative 3D electron microscopy has changed this interpretation. In Xenopus melanophores, Chang et al. showed that VIFs form dynamic cage‐like networks around melanosomes and modulate organelle movement, positioning, and coordinated transport [106]. Together with subsequent work, these findings support a broader role for VIFs as dynamic organizers of membrane organelles, acting through direct interactions mediated by conserved vimentin domains as well as indirect coupling via cytolinker proteins and motor‐driven transport.

In our earlier review [76], we highlighted linkers such as plectin [107, 108], fimbrin [109], and epiplakin [110], which mediate crosstalk between VIFs and other cytoskeletal systems. These cytolinkers contributes to VIFs /IFs network positioning and organization [111, 112, 113]. For example, plectin can link VIF to microtubule tracks in kinesin dependent manner [114]. The perinuclear vimentin physically couples to the nuclear envelope through the LINC complex, particularly via nesprin‐3 and plectin [115, 116]. This coupling preserves nuclear integrity under mechanical stress, stabilizes nuclear positioning, and transmits cytoskeletal forces to the nuclear interior [117, 118]. By reshaping nuclear morphology and influencing chromatin organization, VIFs extend their role from structural support to regulation of gene expression and metabolic activity [119, 120]. In addition, vimentin‐dependent nuclear positioning is essential for maintaining front–rear polarity of the cell and supports efficient cell migration, particularly in confined environments [121].

Among the best‐characterized VIF‐organelle interactions are those with mitochondria. Vimentin associates with mitochondria both indirectly through plectin isoform 1b [122] and directly through residues 41–94 of its N‐terminal head domain [123]. These dual mechanisms allow VIFs to stabilize mitochondria and regulate their motility along MTs [124, 125]. Phosphorylation of the head domain possibly acts as a molecular switch, modulating transient binding. Functionally, these interactions could affect mitochondrial morphology, fission–fusion dynamics, and bioenergetics, linking VIF to metabolism and stress responses.

VIFs also regulate the endoplasmic reticulum (ER). RNF26, an ER‐anchored ubiquitin ligase, binds the vimentin C‐terminal tail, anchoring ER membranes to the IF cytoskeleton [126, 127]. This interaction stabilizes ER continuity in the crowded cytoplasmic regions and supports contacts with mitochondria, endosomes, and the plasma membrane. Such regulation enables dynamic ER remodeling during stress and contributes to cellular homeostasis.

Additional roles include maintaining Golgi cohesion, as vimentin depletion fragments the ribbon‐like Golgi into dispersed ministacks [128]. In adipocytes, vimentin encases lipid droplets via binding to perilipin [129], stabilizing droplet–ER contacts and promoting lipid storage [130]. Beyond mitochondria, ER, and Golgi [131], VIFs influence lysosome, autophagosome and endosome distribution [132]. Collectively, these interactions situate VIF at the crossroads of energy storage, intracellular trafficking, and metabolic regulation. Moreover, single‐particle tracking reveals striking parallels between motility of individual VIF and organelles, with both exhibiting saltatory movement, directional reversals, and pauses—features that underscore their coupled transport and interdependent redistribution.

The shift from viewing VIFs as passive structural cages to recognizing them as dynamic scaffolds has broadened our understanding of their functional diversity within cells. Rather than a mechanical scaffold, VIFs act as responsive platforms that (1) directly engage organelles, (2) tether them via cytolinkers such as plectin, RNF26, and perilipin, and (3) couple with motor‐driven transport to coordinate the spatial distribution of both filaments and organelles. Through these mechanisms, VIFs integrate cytoskeletal architecture with intracellular logistics, adapting organelle positioning to the changing demands of signaling, metabolism, and migration.

## 8 Hybrid Transport Model of VIF Dynamics

VIFs display a remarkable duality of stability and dynamics: they form long‐lived networks yet undergo rapid reorganization throughout the cytoplasm in response to cellular cues and mechanical demands [11, 36, 92, 133, 134]. These dynamics encompass saltatory motility, directional reversals, and localized remodeling [48, 76, 82]. Yet VIF transport is largely microtubule‐dependent, even though motorbased transport typically requires more sustained filament–microtubule engagement than is observed in cells [75]. Building on evidence from multiple studies demonstrating interactions between VIFs, organelles, cytolinker proteins, and molecular motors, we propose a hybrid transport framework (Figure 3) that integrates organelle‐coupled motion, adaptor‐mediated engagement, and local cytoskeletal constraints to reconcile this apparent paradox.

- Intermittent contact sites: Sparse, transient interactions between VIFs and MTs serve as dynamic hotspots of motor‐driven transport. These contacts enable localized translocation without destabilizing the entire network.
- Cytolinker‐mediated transport: Cytolinker proteins, such as plectin, may transiently couple VIFs to motor complexes, enabling selective repositioning of filament segments while preserving overall network continuity and mechanical integrity.
- Organelle‐coupled movement: Membrane‐bound organelles transported by molecular motors can carry associated VIFs. This explains the shared stop‐and‐go motility patterns of VIF and organelles.

Together, these mechanisms reconcile the paradox of sparse filament–microtubule alignment with strong microtubule dependence and position VIFs as dynamic integrators of cytoskeletal and organelle‐based transport. Steady‐state VIF organization likely reflects a balance between directed, organelle‐coupled transport and local retention through cytolinkers, anchoring sites, and network assembly–disassembly, such that dynamic repositioning occurs without net redistribution of the filament network. By enabling context‐dependent, localized repositioning rather than uniform filament translocation, this hybrid framework provides a plausible basis for maintaining both the spatial organization and functional adaptability of the VIF network.

## 9 Future Perspectives

The principles governing VIF filament assembly, structural polymorphism, network organization, dynamics, and interactions with molecular motors and cytolinkers require further investigation. Current mechanistic insights about VIF assembly significantly come from in vitro (cell‐free) assembly studies. In the cellular milieu, how local factors—including interacting proteins, ionic composition, and spatiotemporal cellular demands—collectively govern VIF assembly and dynamics remains poorly understood. The transition from soluble tetramers to short filaments, and their subsequent maturation into extended VIF networks, warrants detailed study in living cells. Recent observations raise the possibility that VIF assembly takes place via phase separation/biocondensates [73, 96]. Further, the IF/VIF system has been proposed to adopt a micelle‐like tubular organization, a perspective that provides a conceptual framework for reconciling hallmark VIF behaviors‐ such as severing, annealing, and dynamic remodeling—with their emergent material properties [52, 55]. This phase‐separation model raises the question of whether distinct polymorphic vimentin tetramer species can co‐assemble or phase‐separate together in vivo. Current structural models of VIFs suggest that the spatial disposition of the tail domain within mature filaments influences the accessibility and regulation of the head domain, including its phosphorylation [55]. This structural coupling between tail and head domains has important signaling implications and raises several questions: how head domains engage organelles such as mitochondria, how tail‐domain positioning modulates these interactions, and how kinases or other regulatory enzymes gain access to their substrates within the filament context.

The molecular basis of VIF interactions with motor proteins remains unresolved, including whether these interactions are direct or adaptor‐mediated. Defining these mechanisms will be essential for understanding how VIF dynamics are regulated within specific cellular regions and may involve cooperative or sequential engagement of multiple interacting partners to bias localized transport.

A major limitation in addressing these questions has been the lack of robust perturbation strategies to selectively depolymerize vimentin filaments or disrupt VIF interactions with motors or organelles. The recently reported FilaBuster [85] platform provides rapid, specific, and spatiotemporally precise disassembly of IF networks, enabling localized or global disruption of IFs while preserving other cytoskeletal systems although its broader applicability and limitations require further validation.

Such acute perturbations offer a unique opportunity to directly interrogate VIF assembly and maintenance by revealing intermediate states of filament disassembly that are otherwise difficult to access. When coupled with high‐resolution live‐cell imaging, FilaBuster could enable direct visualization and tracking of disassembling VIF segments, allowing quantitative analysis of how localized filament loss impacts cytoskeletal architecture, organelle positioning, and force transmission. Integration of these approaches with the extensive body of work on VIF post‐translational modifications may further provide a “top‐down” route to dissect how specific regulatory cues modulate VIF assembly, network remodeling, and functional engagement with motors and organelles.

The continued development of tools like FilaBuster—together with complementary strategies such as vimentin‐targeting small molecules—could provide powerful means to dissect how localized VIF perturbations impact cytoskeletal architecture, organelle positioning, and cell mechanics. Applying these approaches in combination with advanced imaging and quantitative analysis will be particularly informative for understanding how dysregulation of VIF organization contributes to pathological states, including cancer progression and neurodegenerative disease.

## 10 Conclusions

Seminal studies and reviews have progressively redefined intermediate filaments—and vimentin intermediate filaments in particular—from simple mechanical reinforcements to central cytoplasmic integrators that coordinate signaling, organelle organization, and cellular behavior [27, 30, 99, 120, 135, 136, 137]. This conceptual shift positioned IFs as active modulators of cellular architecture, integrating mechanical resilience with dynamic regulation of processes such as cell shape, adhesion, migration, and stress responses. Building on this framework, recent advances in live‐cell imaging, super‐resolution microscopy, and minimally perturbative labeling strategies now enable the visualization of IF networks at unprecedented spatiotemporal resolution. These approaches reveal that IF/VIF networks are intrinsically dynamic polymers whose organization, turnover, and redistribution are tightly coupled to cellular context. Viewing IFs through this dynamic lens is therefore not merely additive but transformative, offering a mechanistic framework for understanding how this filament system unites mechanical stability with active control of intracellular organization and diverse cellular functions.

## Author Contributions

Bhuvanasundar Renganathan (BR): conceptualized the study; wrote and edited the manuscript; prepared the figures. Stephen A. Adam (SA): reviewed the manuscript. Vladimir I. Gelfand (VG): reviewed the manuscript and acquired funding.

## Conflicts of Interest

All authors declare no conflict of interest.

## Data Availability

The data that support the findings of this study are available from the corresponding author upon reasonable request.

## References

[1] L. Pradeau‐Phélut and S. Etienne‐Manneville , “Cytoskeletal Crosstalk: A Focus on Intermediate filaments,” Current Opinion in Cell Biology 87 (2024): 102325, 10.1016/J.CEB.2024.102325. 38359728

[2] F. Huber , A. Boire , M. P. López , and G. H. Koenderink , “Cytoskeletal Crosstalk: When Three Different Personalities Team up,” Current Opinion in Cell Biology 32 (2015): 39–47, 10.1016/J.CEB.2014.10.005. 25460780

[3] K. Barlan and V. I. Gelfand , “Microtubule‐Based Transport and the Distribution, Tethering, and Organization of Organelles,” Cold Spring Harbor Perspectives in Biology 9, no. 5 (2017): a025817, 10.1101/cshperspect.a025817. 28461574 PMC5411697

[4] J. Aureille , S. S. Prabhu , S. F. Barnett , et al., “Focal Adhesions Are Controlled by Microtubules Through Local Contractility Regulation,” The EMBO Journal 43, no. 13 (2024): 2715–2732, 10.1038/s44318-024-00114-4. 38769437 PMC11217342

[5] A. C. Callan‐Jones and R. Voituriez , “Actin Flows in Cell Migration: From Locomotion and Polarity to Trajectories,” Current Opinion in Cell Biology 38 (2016): 12–17, 10.1016/J.CEB.2016.01.003. 26827283

[6] S. Etienne‐Manneville , “Actin and Microtubules in Cell Motility: Which One Is in Control?,” Traffic (Copenhagen, Denmark) 5, no. 7 (2004): 470–477, 10.1111/j.1600-0854.2004.00196.x. 15180824

[7] T. K. Akhshi , D. Wernike , and A. Piekny , “Microtubules and Actin Crosstalk in Cell Migration and Division,” Cytoskeleton 71, no. 1 (2014): 1–23, 10.1002/cm.21150. 24127246

[8] J. Lowery , E. R. Kuczmarski , H. Herrmann , and R. D. Goldman , “Intermediate Filaments Play a Pivotal Role in Regulating Cell Architecture and Function,” The Journal of Biological Chemistry 290, no. 28 (2015): 17145–17153, 10.1074/jbc.R115.640359. 25957409 PMC4498054

[9] J. Ivaska , H. M. Pallari , J. Nevo , and J. E. Eriksson , “Novel Functions of vimentin in Cell Adhesion, Migration, and Signaling,” Experimental Cell Research 313, no. 10 (2007): 2050–2062, 10.1016/J.YEXCR.2007.03.040. 17512929

[10] A. S. Menko , B. M. Bleaken , A. A. Libowitz , L. Zhang , M. A. Stepp , and J. L. Walker , “A central Role for vimentin in Regulating Repair Function During Healing of the Lens Epithelium,” Molecular Biology of the Cell 25, no. 6 (2014): 776–790, 10.1091/mbc.e12-12-0900. 24478454 PMC3952848

[11] L. Kreplak and D. Fudge , “Biomechanical Properties of Intermediate filaments: From Tissues to Single filaments and Back,” BioEssays 29, no. 1 (2007): 26–35, 10.1002/bies.20514. 17187357

[12] H. Ishikawa , R. Bischoff , and H. Holtzer , “Mitosis and Intermediate‐sized filaments in Developing Skeletal Muscle,” The Journal of Cell Biology 38, no. 3 (1968): 538–555, 10.1083/jcb.38.3.538. 5664223 PMC2108373

[13] Y. Uehara , G. R. Campbell , and G. Burnstock , “Cytoplasmic filaments in Developing and Adult Vertebrate Smooth Muscle,” Journal of Cell Biology 50, no. 2 (1971): 484–497, 10.1083/jcb.50.2.484. 5165265 PMC2108268

[14] P. M. Steinert and D. R. Roop , “Molecular and Cellular Biology of Intermediate filaments,” Annual Review of Biochemistry 57 (1988): 593–625, 10.1146/annurev.bi.57.070188.003113. 3052284

[15] J. V. Small and A. Sobieszek , “Studies on the Function and Composition of the 10‐NM(100‐A) filaments of Vertebrate Smooth Muscle,” Journal of Cell Science 23 (1977): 243–268, 10.1242/jcs.23.1.243. 561084

[16] P. M. Steinert , S. B. Zimmerman , J. M. Starger , and R. D. Goldman , “Ten‐nanometer filaments of Hamster BHK‐21 Cells and Epidermal Keratin filaments Have Similar Structures,” Proceedings of the National Academy of Sciences 75, no. 12 (1978): 6098–6101, 10.1073/pnas.75.12.6098. PMC393125 282627

[17] A. Bignami , L. F. Eng , D. Dahl , and C. T. Uyeda , “Localization of the Glial Fibrillary Acidic Protein in Astrocytes by Immunofluorescence,” Brain Research 43, no. 2 (1972): 429–435, 10.1016/0006-8993(72)90398-8. 4559710

[18] R. G. Oshima , “Intermediate filaments: A Historical Perspective,” Experimental Cell Research 313, no. 10 (2007): 1981–1994, 10.1016/j.yexcr.2007.04.007. 17493611 PMC1950476

[19] E. Lazarides , “Intermediate filaments: A Chemically Heterogeneous, Developmentally Regulated Class of Proteins,” Annual Reviews 51 (1982): 219–250, 10.1146/annurev.bi.51.070182.001251. 6180679

[20] W. W. Franke , E. Schmid , M. Osborn , and K. Weber , “Different Intermediate‐sized filaments Distinguished by Immunofluorescence Microscopy,” Proceedings of the National Academy of Sciences 75, no. 10 (1978): 5034–5038, 10.1073/pnas.75.10.5034. PMC336257 368806

[21] G. S. Bennett , S. A. Fellini , J. M. Croop , J. J. Otto , J. Bryan , and H. Holtzer , “Differences Among 100‐A Filamentilament Subunits From Different Cell Types,” Proceedings of the National Academy of Sciences of the United States of America 75, no. 9 (1978): 4364–4368, 10.1073/pnas.75.9.4364. 360218 PMC336115

[22] P. M. Steinert , W. W. Idler , and R. D. Goldman , “Intermediate filaments of Baby Hamster Kidney (BHK‐21) Cells and Bovine Epidermal Keratinocytes Have Similar Ultrastructures and Subunit Domain Structures,” Proceedings of the National Academy of Sciences of the United States of America 77, no. 8 (1980): 4534–4538, 10.1073/pnas.77.8.4534. 6159631 PMC349878

[23] P. M. Steinert , W. W. Idler , F. Cabral , M. M. Gottesman , and R. D. Goldman , “In Vitro Assembly of Homopolymer and Copolymer filaments From Intermediate Filament Subunits of Muscle and Fibroblastic Cells,” Proceedings of the National Academy of Sciences of the United States of America 78, no. 6 I (1981): 3692–3696, 10.1073/pnas.78.6.3692. 6943573 PMC319637

[24] R. V. Zackroff and R. D. Goldman , “In Vitro Assembly of Intermediate filaments From Baby Hamster Kidney (BHK‐21) Cells,” Proceedings of the National Academy of Sciences of the United States of America 76, no. 12 (1979): 6226–6230, 10.1073/pnas.76.12.6226. 293716 PMC411836

[25] P. M. Steinert , A. C. Steven , and D. R. Roop , “The Molecular Biology of Intermediate filaments,” Cell 42, no. 2 (1985): 411–419, 10.1016/0092-8674(85)90098-4. 2411418

[26] H. Herrmann , M. Häner , M. Brettel , et al., “Structure and Assembly Properties of the Intermediate Filament Protein Vimentin: The Role of Its Head, Rod and Tail Domains,” Journal of Molecular Biology 264, no. 5 (1996): 933–953, 10.1006/jmbi.1996.0688. 9000622

[27] H. Herrmann , H. Bär , L. Kreplak , S. V. Strelkov , and U. Aebi , “Intermediate filaments: From Cell Architecture to Nanomechanics,” Nature Reviews Molecular Cell Biology 8, no. 7 (2007): 562–573, 10.1038/nrm2197. 17551517

[28] J. E. Eriksson , T. Dechat , B. Grin , et al., “Introducing Intermediate filaments: From Discovery to Disease,” Journal of Clinical Investigation 119, no. 7 (2009): 1763–1771, 10.1172/JCI38339. 19587451 PMC2701876

[29] E. Colucci‐Guyon , M.‐M. Portier , I. Dunia , D. Paulin , S. Pournin , and C. Babinet , “Mice Lacking Vimentin Develop and Reproduce Without an Obvious Phenotype,” Cell 79, no. 4 (1994): 679–694, 10.1016/0092-8674(94)90553-3. 7954832

[30] K. M. Ridge , J. E. Eriksson , M. Pekny , and R. D. Goldman , “Roles of vimentin in Health and Disease,” Genes and Development 36 (2022): 391–407, 10.1101/gad.349358.122. 35487686 PMC9067405

[31] P. Soellner , R. A. Quinlan , and W. W. Franke , “Identification of a Distinct Soluble Subunit of an Intermediate Filament Protein: Tetrameric Vimentin From Living Cells,” Proceedings of the National Academy of Sciences of the United States of America 82, no. 23 (1985): 7929–7933, 10.1073/pnas.82.23.7929. 3865206 PMC390883

[32] H. Herrmann , M. Häner , M. Brettel , N. O. Ku , and U. Aebi , “Characterization of Distinct Early Assembly Units of Different Intermediate Filament Proteins,” Journal of Molecular Biology 286, no. 5 (1999): 1403–1420, 10.1006/jmbi.1999.2528. 10064706

[33] J. E. Eriksson , P. Opal , and R. D. Goldman , “Intermediate Filament Dynamics,” Current Opinion in Cell Biology 4, no. 1 (1992): 99–104, 10.1016/0955-0674(92)90065-K. 1558758

[34] K. J. Angelides , K. E. Smith , and M. Takeda , “Assembly and Exchange of Intermediate Filament Proteins of Neurons: Neurofilaments Are Dynamic Structures,” The Journal of Cell Biology 108, no. 4 (1989): 1495–1506, 10.1083/jcb.108.4.1495. 2925792 PMC2115529

[35] Y. Nakamura , M. Takeda , K. J. Angelides , K. Tada , S. Hariguchi , and T. Nishimura , “Assembly, Disassembly, and Exchange of Glial Fibrillary Acidic Protein,” Glia 4, no. 1 (1991): 101–110, 10.1002/GLIA.440040112. 1828780

[36] G. Çolakoğlu and A. Brown , “Intermediate filaments Exchange Subunits Along Their Length and Elongate by End‐to‐end Annealing,” Journal of Cell Biology 185, no. 5 (2009): 769–777, 10.1083/jcb.200809166. 19468066 PMC2711597

[37] T. R. Coleman and E. Lazarides , “Continuous Growth of Vimentin filaments in Mouse Fibroblasts,” Journal of Cell Science 103, no. 3 (1992): 689–698, 10.1242/jcs.103.3.689. 1478965

[38] J. Ngai , T. R. Coleman , and E. Lazarides , “Localization of Newly Synthesized Vimentin Subunits Reveals a Novel Mechanism of Intermediate Filament Assembly,” Cell 60, no. 3 (1990): 415–427, 10.1016/0092-8674(90)90593-4. 2406021

[39] J. M. Starger , W. E. Brown , A. E. Goldman , and R. D. Goldman , “Biochemical and Immunological Analysis of Rapidly Purified 10‐nm filaments From Baby Hamster Kidney (BHK‐21) Cells,” Journal of Cell Biology 78, no. 1 (1978): 93–109, 10.1083/jcb.78.1.93. 566763 PMC2110160

[40] H. Herrmann and U. Aebi , “Intermediate Filaments: Molecular Structure, Assembly Mechanism, and Integration Into Functionally Distinct Intracellular Scaffolds,” Annual Review of Biochemistry 73 (2004): 749–789, 10.1146/annurev.biochem.73.011303.073823. 15189158

[41] T. Nebl , K. N. Pestonjamasp , J. D. Leszyk , J. L. Crowley , S. W. Oh , and E. J. Luna , “Proteomic Analysis of a Detergent‐resistant Membrane Skeleton From Neutrophil Plasma Membranes,” Journal of Biological Chemistry 277, no. 45 (2002): 43399–43409, 10.1074/JBC.M205386200/ATTACHMENT/628D082A-F8C9-49D8-B49C-E7A92472E497/MMC1.PDF. 12202484

[42] F. Castro‐Muñozledo , D. G. Meza‐Aguilar , R. Domínguez‐Castillo , V. Hernández‐Zequinely , and E. Sánchez‐Guzmán , “Vimentin as a Marker of Early Differentiating, Highly Motile Corneal Epithelial Cells,” Journal of Cellular Physiology 232, no. 4 (2017): 818–830, 10.1002/jcp.25487. 27404216

[43] S. Pattabiraman , G. K. Azad , T. Amen , et al., “Vimentin Protects Differentiating Stem Cells From Stress,” Scientific Reports 10, no. 1 (2020): 1–15, 10.1038/s41598-020-76076-4. 33177544 PMC7658978

[44] T. A. Smith , S. V. Strelkov , P. Burkhard , U. Aebi , and D. A. D. Parry , “Sequence Comparisons of Intermediate Filament Chains: Evidence of a Unique Functional/Structural Role for Coiled‐coil Segment 1A and Linker L1,” Journal of Structural Biology 137, no. 1–2 (2002): 128–145, 10.1006/jsbi.2002.4438. 12064940

[45] S. V. Strelkov , H. Herrmann , and U. Aebi , “Molecular Architecture of Intermediate filaments,” BioEssays 25, no. 3 (2003): 243–251, 10.1002/bies.10246. 12596228

[46] K. Albers and E. Fuchs , “The Molecular Biology of Intermediate Filament Proteins,” International Review of Cytology 134, no. 7 (1992): 243–279, 10.1016/s0074-7696(08)62030-6. 1374743

[47] S. Nicolet , H. Herrmann , U. Aebi , and S. V. Strelkov , “Atomic Structure of Vimentin Coil 2,” Journal of Structural Biology 170, no. 2 (2010): 369–376, 10.1016/j.jsb.2010.02.012. 20176112

[48] M. Yoon , R. D. Moir , V. Prahlad , and R. D. Goldman , “Motile Properties of vimentin Intermediate Filament Networks in Living Cells,” Journal of Cell Biology 143, no. 1 (1998): 147–157, 10.1083/jcb.143.1.147. 9763427 PMC2132819

[49] S. Sivagurunathan , A. Vahabikashi , H. Yang , et al., “Expression of Vimentin Alters Cell Mechanics, Cell–Cell Adhesion, and Gene Expression Profiles Suggesting the Induction of a Hybrid EMT in human Mammary Epithelial Cells,” Frontiers in Cell and Developmental Biology 10 (2022), 10.3389/fcell.2022.929495. PMC9527304 36200046

[50] W. W. Franke , E. Schmid , C. Grund , and B. Geiger , “Intermediate Filament Proteins in Nonfilamentous Structures: Transient Disintegration and Inclusion of Subunit Proteins in Granular Aggregates,” Cell 30, no. 1 (1982): 103–113, 10.1016/0092-8674(82)90016-2. 6751555

[51] P. J. Vermeire , A. V. Lilina , H. M. Hashim , et al., “Molecular Structure of Soluble Vimentin Tetramers,” Scientific Reports 13, no. 1 (2023): 1–16, 10.1038/s41598-023-34814-4. 37258554 PMC10232555

[52] S. Jeong and N.‐C. Ha , “Deciphering Vimentin Assembly: Bridging Theoretical Models and Experimental Approaches,” Molecules and Cells 47, no. 7 (2024): 100080, 10.1016/j.mocell.2024.100080. 38871297 PMC11267000

[53] N. Mücke , T. Wedig , A. Bürer , et al., “Molecular and Biophysical Characterization of Assembly‐starter Units of human Vimentin,” Journal of Molecular Biology 340, no. 1 (2004): 97–114, 10.1016/j.jmb.2004.04.039. 15184025

[54] H. Herrmann , S. V. Strelkov , B. Feja , et al., “The Intermediate Filament Protein Consensus Motif of Helix 2B: Its Atomic Structure and Contribution to Assembly,” Journal of Molecular Biology 298, no. 5 (2000): 817–832, 10.1006/jmbi.2000.3719. 10801351

[55] M. Eibauer , M. S. Weber , R. Kronenberg‐Tenga , et al., “Vimentin filaments Integrate Low‐complexity Domains in a Complex Helical Structure,” Nature Structural & Molecular Biology 31, no. 6 (2024): 939–949, 10.1038/s41594-024-01261-2. PMC11189308 38632361

[56] F. N. Vicente , M. Lelek , J. Y. Tinevez , et al., “Molecular Organization and Mechanics of Single Vimentin filaments Revealed by Super‐resolution Imaging,” Science Advances 8, no. 8 (2022): 2696, 10.1126/sciadv.abm2696. PMC8880768 35213220

[57] P. M. Steinert , L. N. Marekov , and D. A. D. Parry , “Diversity of Intermediate Filament Structure: Evidence That the Alignment of Coiled‐coil Molecules in Vimentin Is Different From That in Keratin Intermediate filaments,” Journal of Biological Chemistry 268, no. 33 (1993): 24916–24925, 10.1016/s0021-9258(19)74552-9. 7693709

[58] D. A. D. Parry and P. M. Steinert , “Intermediate filaments: Molecular Architecture, Assembly, Dynamics and Polymorphism,” Quarterly Reviews of Biophysics 32, no. 2 (1999): 99–187, 10.1017/S0033583500003516. 10845237

[59] N. T. Snider and M. B. Omary , “Post‐translational Modifications of Intermediate Filament Proteins: Mechanisms and Functions,” Nature Reviews Molecular Cell Biology 15 (2014): 163–177, 10.1038/nrm3753. 24556839 PMC4079540

[60] R. K. Sihag , M. Inagaki , T. Yamaguchi , T. B. Shea , and H. C. Pant , “Role of Phosphorylation on the Structural Dynamics and Function of Types III and IV Intermediate filaments,” Academic Press 313 (2007): 2098–2109, 10.1016/j.yexcr.2007.04.010. PMC2570114 17498690

[61] H. Inada , H. Togashi , Y. Nakamura , K. Kaibuchi , K. I. Nagata , and M. Inagaki , “Balance Between Activities of Rho Kinase and Type 1 Protein Phosphatase Modulates Turnover of Phosphorylation and Dynamics of Desmin/Vimentin filaments,” Journal of Biological Chemistry 274, no. 49 (1999): 34932–34939, 10.1074/jbc.274.49.34932. 10574968

[62] B. T. Helfand , M. G. Mendez , S. N. P. Murthy , et al., “Vimentin Organization Modulates the Formation of Lamellipodia,” Molecular Biology of the Cell 22, no. 8 (2011): 1274–1289, 10.1091/MBC.E10-08-0699/ASSET/IMAGES/LARGE/1274FIG10.JPEG. 21346197 PMC3078081

[63] C. Li , F. P. McManus , C. Plutoni , et al., “Quantitative SUMO Proteomics Identifies PIAS1 Substrates Involved in Cell Migration and Motility,” Nature Communications 11, no. 1 (2020): 1–14, 10.1038/s41467-020-14581-w. PMC7012886 32047143

[64] J. E. Eriksson , T. He , A. V. Trejo‐Skalli , et al., “Specific in Vivo Phosphorylation Sites Determine the Assembly Dynamics of Vimentin Intermediate filaments,” Journal of Cell Science 117, no. 6 (2004): 919–932, 10.1242/JCS.00906. 14762106

[65] M. B. Omary , N. O. Ku , G. Z. Tao , D. M. Toivola , and J. Liao , “Heads and Tails″ of Intermediate Filament Phosphorylation: Multiple Sites and Functional Insights,” Trends in Biochemical Sciences 31, no. 7 (2006): 383–394, 10.1016/j.tibs.2006.05.008. 16782342

[66] H. J. Tarbet , L. Dolat , T. J. Smith , et al., “Site‐specific Glycosylation Regulates the Form and Function of the Intermediate Filament Cytoskeleton,” Elife 7 (2018): 65–72, 10.7554/eLife.31807. PMC5841932 29513221

[67] Z. Wang , A. Pandey , and G. W. Hart , “Dynamic Interplay Between O‐linked N‐acetylglucosaminylation and Glycogen Synthase Kinase‐3‐dependent Phosphorylation,” Molecular and Cellular Proteomics 6, no. 8 (2007): 1365–1379, 10.1074/mcp.M600453-MCP200. 17507370

[68] W. Ip , M. K. Hartzer , Y. Y. S. Pang , and R. M. Robson , “Assembly of Vimentin in Vitro and Its Implications Concerning the Structure of Intermediate filaments,” Journal of Molecular Biology 183, no. 3 (1985): 365–375, 10.1016/0022-2836(85)90007-5. 4040578

[69] N. Mücke , L. Kämmerer , S. Winheim , et al., “Assembly Kinetics of Vimentin Tetramers to Unit‐Length Filaments: A Stopped‐Flow Study,” Biophysical Journal 114, no. 10 (2018): 2408–2418, 10.1016/J.BPJ.2018.04.032. 29754715 PMC6129470

[70] X. Zhou , Y. Lin , M. Kato , et al., “Transiently Structured Head Domains Control Intermediate Filament Assembly,” Proceedings of the National Academy of Sciences of the United States of America 118, no. 8 (2021): 2022121118, 10.1073/pnas.2022121118. PMC7923618 33593918

[71] D. A. D. Parry , S. V. Strelkov , P. Burkhard , U. Aebi , and H. Herrmann , “Towards a Molecular Description of Intermediate Filament Structure and Assembly,” Academic Press 313 (2007): 2204–2216, 10.1016/j.yexcr.2007.04.009. 17521629

[72] D. Pérez‐Sala and S. Zorrilla , “Versatility of Vimentin Assemblies: From filaments to Biomolecular Condensates and Back,” European Journal of Cell Biology 104, no. 2 (2025): 151487, 10.1016/j.ejcb.2025.151487. 40194320

[73] P. Martínez‐Cenalmor , A. E. Martínez , D. Moneo‐Corcuera , P. González‐Jiménez , and D. Pérez‐Sala , “Oxidative Stress Elicits the Remodeling of Vimentin filaments Into Biomolecular Condensates,” Redox Biology 75 (2024): 103282, 10.1016/j.redox.2024.103282. 39079387 PMC11338992

[74] A. H. Sharpe , L. B. Chen , J. R. Murphy , and B. N. Fields , “Specific Disruption of Vimentin Filament Organization in Monkey Kidney CV‐1 Cells by Diphtheria Toxin, Exotoxin A, and Cycloheximide,” Proceedings of the National Academy of Sciences of the United States of America 77, no. 12 II (1980): 7267–7271, 10.1073/pnas.77.12.7267. 6784120 PMC350483

[75] E. H. Ball and S. J. Singer , “Association of Microtubules and Intermediate filaments in Normal Fibroblasts and Its Disruption Upon Transformation by a Temperature‐sensitive Mutant of Rous sarcoma Virus,” Proceedings of the National Academy of Sciences of the United States of America 78, no. 11 II (1981): 6986–6990, 10.1073/pnas.78.11.6986. 6273900 PMC349178

[76] A. Robert , C. Hookway , and V. I. Gelfand , “Intermediate Filament Dynamics: What We Can See Now and Why It Matters,” BioEssays : News and Reviews in Molecular, Cellular and Developmental Biology 38, no. 3 (2016): 232–243, 10.1002/bies.201500142. 26763143 PMC4772765

[77] V. Prahlad , M. Yoon , R. D. Moir , R. D. Vale , and R. D. Goldman , “Rapid Movements of Vimentin on Microtubule Tracks: Kinesin‐dependent Assembly of Intermediate Filament Networks,” The Journal of Cell Biology 143, no. 1 (1998): 159–170, 10.1083/jcb.143.1.159. 9763428 PMC2132817

[78] A. Robert , H. Herrmann , M. W. Davidson , and V. I. Gelfand , “Microtubule‐dependent Transport of Vimentin Filament Precursors Is Regulated by Actin and by the Concerted Action of Rho‐ and p21‐activated Kinases,” FASEB Journal 28, no. 7 (2014): 2879–2890, 10.1096/fj.14-250019. 24652946 PMC4062827

[79] B. Renganathan , J. P. Zewe , Y. Cheng , et al., “Gigaxonin Is Required for Intermediate Filament Transport,” FASEB Journal 37, no. 5 (2023): 22886, 10.1096/fj.202202119R. PMC10237250 37043392

[80] S. Winheim , A. R. Hieb , M. Silbermann , et al., “Deconstructing the Late Phase of Vimentin Assembly by Total Internal Reflection Fluorescence Microscopy (TIRFM),” PLoS ONE 6, no. 4 (2011): 19202, 10.1371/journal.pone.0019202. PMC3081349 21544245

[81] C. Hookway , L. Ding , M. W. Davidson , J. Z. Rappoport , G. Danuser , and V. I. Gelfand , “Microtubule‐dependent Transport and Dynamics of Vimentin Intermediate filaments,” Molecular Biology of the Cell 26, no. 9 (2015): 1675–1686, 10.1091/mbc.E14-09-1398. 25717187 PMC4436779

[82] B. Renganathan , A. S. Moore , W. H. Yeo , et al., “Vimentin Filament Transport and Organization Revealed by Single‐particle Tracking and 3D FIB‐SEM,” The Journal of Cell Biology 224, no. 4 (2025), 10.1083/jcb.202406054. PMC11893169 40062969

[83] L. Wang and A. Brown , “Rapid Intermittent Movement of Axonal Neurofilaments Observed by Fluorescence Photobleaching,” Molecular Biology of the Cell 12, no. 10 (2001): 3257, 10.1091/MBC.12.10.3257. 11598207 PMC60171

[84] S. Roy , P. Coffee , G. Smith , R. K. H. Liem , S. T. Brady , and M. M. Black , “Neurofilaments Are Transported Rapidly but Intermittently in Axons: Implications for Slow Axonal Transport,” Journal of Neuroscience 20, no. 18 (2000): 6849–6861, 10.1523/jneurosci.20-18-06849.2000. 10995829 PMC6772820

[85] A. S. Moore , T. Krug , S. B. Hansen , et al., “FilaBuster: A Strategy for Rapid, Specific, and Spatiotemporally Controlled Intermediate Filament Disassembly,” BioRxiv (2025), 10.1101/2025.04.20.649718.

[86] G. Dutour‐Provenzano and S. Etienne‐Manneville , “Intermediate filaments,” Current Biology 31, no. 10 (2021): R522–R529, 10.1016/j.cub.2021.04.011. 34033784

[87] R. D. Goldman , “The Role of Three Cytoplasmic Fibers in BHK‐21 Cell Motility: I. Microtubules and the Effects of Colchicine,” Journal of Cell Biology 51, no. 3 (1971): 752–762, 10.1083/jcb.51.3.752. 4942774 PMC2108053

[88] F. K. Gyoeva and V. I. Gelfand , “Coalignment of Vimentin Intermediate filaments With Microtubules Depends on Kinesin,” Nature 353, no. 6343 (1991): 445–448, 10.1038/353445a0. 1832745

[89] B. T. Helfand , A. Mikami , R. B. Vallee , and R. D. Goldman , “A Requirement for Cytoplasmic Dynein and Dynactin in Intermediate Filament Network Assembly and Organization,” Journal of Cell Biology 157, no. 5 (2002): 795–806, 10.1083/jcb.200202027. 12034772 PMC2173407

[90] E. J. Clarke and V. Allan , “Intermediate filaments: Vimentin Moves in,” Current Biology 12, no. 17 (2002): R596–R598, 10.1016/S0960-9822(02)01102-8. 12225682

[91] L. Schaedel , C. Lorenz , A. V. Schepers , S. Klumpp , and S. Köster , “Vimentin Intermediate filaments Stabilize Dynamic Microtubules by Direct Interactions,” Nature Communications 12, no. 1 (2021): 3799, 10.1038/s41467-021-23523-z. PMC8213705 34145230

[92] Z. Gan , L. Ding , C. J. Burckhardt , et al., “Vimentin Intermediate Filaments Template Microtubule Networks to Enhance Persistence in Cell Polarity and Directed Migration,” Cell Systems 3, no. 3 (2016): 252–263, 10.1016/j.cels.2016.08.007. 27667364 PMC5055390

[93] Y. Jiu , J. Lehtimäki , S. Tojkander , et al., “Bidirectional Interplay Between Vimentin Intermediate Filaments and Contractile Actin Stress Fibers,” Cell Reports 11, no. 10 (2015): 1511–1518, 10.1016/j.celrep.2015.05.008. 26027931

[94] H. Wu , Y. Shen , S. Sivagurunathan , et al., “Vimentin Intermediate filaments and Filamentous Actin Form Unexpected Interpenetrating Networks That Redefine the Cell Cortex,” Proceedings of the National Academy of Sciences 119, no. 10 (2022): 2115217119, 10.1073/pnas.2115217119. PMC8915831 35235449

[95] Y. Shen , H. Wu , P. J. Lu , et al., “Effects of Vimentin Intermediate Filaments on the Structure and Dynamics of in Vitro Multicomponent Interpenetrating Cytoskeletal Networks,” Physical Review Letters 127, no. 10 (2021): 108101, 10.1103/PhysRevLett.127.108101. 34533352 PMC10725302

[96] A. Basu , T. Krug , B. du Pont , et al., “Vimentin Undergoes Liquid–liquid Phase Separation to Form Droplets Which Wet and Stabilize Actin Fibers,” Proceedings of the National Academy of Sciences of the United States of America 122, no. 10 (2025), 10.1073/pnas.2418624122. PMC11912372 40030010

[97] P. C. Marks , B. R. Hewitt , M. A. Baird , G. Wiche , and R. J. Petrie , “Plectin Linkages Are Mechanosensitive and Required for the Nuclear Piston Mechanism of Three‐dimensional Cell Migration,” Molecular Biology of the Cell 33, no. 12 (2022): ar104, 10.1091/mbc.E21-08-0414. 35857713 PMC9635290

[98] N. H. Alami , P. Jung , and A. Brown , “Myosin Va Increases the Efficiency of Neurofilament Transport by Decreasing the Duration of Long‐term Pauses,” Journal of Neuroscience 29, no. 20 (2009): 6625–6634, 10.1523/JNEUROSCI.3829-08.2009. 19458233 PMC2943491

[99] K. Barlan , M. J. Rossow , and V. I. Gelfand , “The Journey of the Organelle: Teamwork and Regulation in Intracellular Transport,” Elsevier Current Trends 25, no. 4 (2013): 483–488, 10.1016/j.ceb.2013.02.018. PMC3723706 23510681

[100] P. A. Janmey , U. Euteneuer , P. Traub , and M. Schliwa , “Viscoelastic Properties of Vimentin Compared With Other Filamentous Biopolymer Networks,” The Journal of Cell Biology 113, no. 1 (1991): 155–160, 10.1083/JCB.113.1.155. 2007620 PMC2288924

[101] F. Alisafaei , K. Mandal , R. Saldanha , et al., “Vimentin Is a Key Regulator of Cell Mechanosensing Through Opposite Actions on Actomyosin and Microtubule Networks,” Communications Biology 7, no. 1 (2024): 658, 10.1038/s42003-024-06366-4. 38811770 PMC11137025

[102] J. P. Conboy , M. G. Lettinga , P. E. Boukany , F. C. MacKintosh , and G. H. Koenderink , “Actin and vimentin Jointly Control Cell Viscoelasticity and Compression Stiffening,” BioRxiv (2025), 10.1101/2025.01.01.630993. PMC12930353 41400926

[103] Y. Jiu , J. Peränen , N. Schaible , et al., “Vimentin Intermediate filaments Control Actin Stress fiber Assembly Through GEF‐H1 and RhoA,” Journal of Cell Science 130, no. 5 (2017): 892–902, 10.1242/jcs.196881. 28096473 PMC5358333

[104] B. Eckes , D. Dogic , E. Colucci‐Guyon , et al., “Impaired Mechanical Stability, Migration and Contractile Capacity in Vimentin Deficient Fibroblasts,” Journal of Cell Science 111, no. 13 (1998): 1897–1907, 10.1242/jcs.111.13.1897. 9625752

[105] W. W. Franke , M. Hergt , and C. Grund , “Rearrangement of the Vimentin Cytoskeleton During Adipose Conversion: Formation of an Intermediate Filament Cage Around Lipid Globules,” Cell 49, no. 1 (1987): 131–141, 10.1016/0092-8674(87)90763-X. 3548999

[106] L. Chang , K. Barlan , Y. H. Chou , et al., “The Dynamic Properties of Intermediate filaments During Organelle Transport,” Journal of Cell Science 122, no. 16 (2009): 2914–2923, 10.1242/jcs.046789. 19638410 PMC2724608

[107] G. Wiche , “Plectin‐Mediated Intermediate Filament Functions: Why Isoforms Matter,” Cells 10, no. 8 (2021): 2154, 10.3390/cells10082154. 34440923 PMC8391331

[108] G. Burgstaller , M. Gregor , L. Winter , and G. Wiche , “Keeping the Vimentin Network Under Control: Cell–Matrix Adhesion–associated Plectin 1f Affects Cell Shape and Polarity of Fibroblasts,” Molecular Biology of the Cell 21, no. 19 (2010): 3362–3375, 10.1091/mbc.e10-02-0094. 20702585 PMC2947472

[109] I. Correia , D. Chu , Y. H. Chou , R. D. Goldman , and P. Matsudaira , “Integrating the Actin and Vimentin CytoskeletonsAdhesion‐Dependent Formation of Fimbrin–Vimentin Complexes in Macrophages,” Journal of Cell Biology 146, no. 4 (1999): 831–842, 10.1083/JCB.146.4.831. 10459017 PMC2156141

[110] W. Wang , H. Sumiyosi , H. Yoshioka , and S. Fujiwara , “Interactions Between Epiplakin and Intermediate filaments,” The Journal of Dermatology 33, no. 8 (2006): 518–527, 10.1111/j.1346-8138.2006.00127.x. 16923132

[111] M. Prechova , Z. Adamova , A.‐L. Schweizer , et al., “Plectin‐mediated Cytoskeletal Crosstalk Controls Cell Tension and Cohesion in Epithelial Sheets,” The Journal of Cell Biology 221, no. 3 (2022), 10.1083/jcb.202105146. PMC8932528 35139142

[112] R. Spurny , M. Gregor , M. J. Castañón , and G. Wiche , “Plectin Deficiency Affects Precursor Formation and Dynamics of Vimentin Networks,” Experimental Cell Research 314, no. 19 (2008): 3570–3580, 10.1016/j.yexcr.2008.09.012. 18848541

[113] R. Windoffer , N. Schwarz , S. Yoon , et al., “Quantitative Mapping of Keratin Networks in 3D,” Elife 11 (2022): 75894, 10.7554/ELIFE.75894. PMC8979588 35179484

[114] R. Bhattacharya , A. M. Gonzalez , P. J. DeBiase , et al., “Recruitment of Vimentin to the Cell Surface by β3 Integrin and Plectin Mediates Adhesion Strength,” Journal of Cell Science 122, no. 9 (2009): 1390–1400, 10.1242/jcs.043042. 19366731 PMC2721003

[115] M. Ketema , M. Kreft , P. Secades , H. Janssen , and A. Sonnenberg , “Nesprin‐3 Connects Plectin and Vimentin to the Nuclear Envelope of Sertoli Cells but Is Not Required for Sertoli Cell Function in Spermatogenesis,” Molecular Biology of the Cell 24, no. 15 (2013): 2454–2466, 10.1091/mbc.E13-02-0100. 23761073 PMC3727937

[116] K. Wilhelmsen , S. H. M. Litjens , I. Kuikman , et al., “Nesprin‐3, a Novel Outer Nuclear Membrane Protein, Associates With the Cytoskeletal Linker Protein Plectin,” The Journal of Cell Biology 171, no. 5 (2005): 799–810, 10.1083/jcb.200506083. 16330710 PMC2171291

[117] M. Ketema and A. Sonnenberg , “Nesprin‐3: A Versatile Connector Between the Nucleus and the Cytoskeleton,” Biochemical Society Transactions 39, no. 6 (2011): 1719–1724, 10.1042/BST20110669. 22103514

[118] A. E. Patteson , A. Vahabikashi , K. Pogoda , et al., “Vimentin Protects Cells Against Nuclear Rupture and DNA Damage During Migration,” The Journal of Cell Biology 218, no. 12 (2019): 4079–4092, 10.1083/jcb.201902046. 31676718 PMC6891099

[119] A. B. Ndiaye , G. H. Koenderink , and M. Shemesh , “Intermediate Filaments in Cellular Mechanoresponsiveness: Mediating Cytoskeletal Crosstalk from Membrane to Nucleus and Back,” 10 (2022), 882037, 10.3389/fcell.2022.882037. PMC9035595 35478961

[120] P. A. Coulombe and P. Wong , “Cytoplasmic Intermediate filaments Revealed as Dynamic and Multipurpose Scaffolds,” Nature Cell Biology 6, no. 8 (2004): 699–706, 10.1038/ncb0804-699. 15303099

[121] R. A. Quinlan , N. Schwarz , R. Windoffer , et al., “A Rim‐and‐spoke Hypothesis to Explain the Biomechanical Roles for Cytoplasmic Intermediate Filament Networks,” Journal of Cell Science 130, no. 20 (2017): 3437–3445, 10.1242/jcs.202168. 29032358 PMC6518161

[122] L. Winter , C. Abrahamsberg , and G. Wiche , “Plectin Isoform 1b Mediates Mitochondrion–intermediate Filament Network Linkage and Controls Organelle Shape,” The Journal of Cell Biology 181, no. 6 (2008): 903–911, 10.1083/jcb.200710151. 18541706 PMC2426950

[123] O. E. Nekrasova , M. G. Mendez , I. S. Chernoivanenko , et al., “Vimentin Intermediate filaments Modulate the Motility of Mitochondria,” Molecular Biology of the Cell 22, no. 13 (2011): 2282–2289, 10.1091/mbc.E10-09-0766. 21562225 PMC3128530

[124] I. B. Alieva , A. S. Shakhov , A. A. Dayal , A. S. Churkina , O. I. Parfenteva , and A. A. Minin , “Unique Role of Vimentin in the Intermediate Filament Proteins Family,” Biochemistry 89, no. 4 (2024): 726–736, 10.1134/S0006297924040114. 38831508

[125] I. S. Chernoivanenko , E. A. Matveeva , V. I. Gelfand , R. D. Goldman , and A. A. Minin , “Mitochondrial Membrane Potential Is Regulated by Vimentin Intermediate filaments,” FASEB Journal 29, no. 3 (2015): 820–827, 10.1096/fj.14-259903. 25404709 PMC4422353

[126] T. Cremer , L. M. Voortman , E. Bos , et al., “RNF26 binds Perinuclear Vimentin filaments to Integrate ER and Endolysosomal Responses to Proteotoxic Stress,” The EMBO Journal 42, no. 18 (2023): 111252, 10.15252/EMBJ.2022111252. PMC10505911 37519262

[127] P. Strzyz , “RNF26 and vimentin Orchestrate ER Stress Recovery,” Nature Reviews Molecular Cell Biology 24, no. 10 (2023): 689–689, 10.1038/s41580-023-00655-2. 37592062

[128] T. Vitali , R. Sanchez‐Alvarez , T. M. Witkos , et al., “Vimentin Intermediate filaments Provide Structural Stability to the Mammalian Golgi Complex,” Journal of Cell Science no. 20 (2023): 136, 10.1242/jcs.260577. PMC10617613 37732478

[129] H. Heid , S. Rickelt , R. Zimbelmann , et al., “On the Formation of Lipid Droplets in human Adipocytes: The Organization of the Perilipin‐vimentin Cortex,” PLoS ONE 9, no. 2 (2014): 90386, 10.1371/journal.pone.0090386. PMC3938729 24587346

[130] J. G. Lieber and R. M. Evans , “Disruption of the vimentin Intermediate Filament System During Adipose Conversion of 3T3‐L1 Cells Inhibits Lipid Droplet Accumulation,” Journal of Cell Science 109, no. 13 (1996): 3047–3058, http://www.ncbi.nlm.nih.gov/pubmed/9004039. 9004039 10.1242/jcs.109.13.3047

[131] Y. S. Gao and E. Sztul , “A Novel Interaction of the Golgi Complex With the Vimentin Intermediate Filament Cytoskeleton,” Journal of Cell Biology 152, no. 5 (2001): 877–894, 10.1083/JCB.152.5.877. 11238446 PMC2198822

[132] O. Biskou , V. Casanova , K. M. Hooper , et al., “The Type III Intermediate Filament Vimentin Regulates Organelle Distribution and Modulates Autophagy,” PLoS ONE 14, no. 1 (2019): 0209665, 10.1371/journal.pone.0209665. PMC6353089 30699149

[133] Q. D. Tran , M. Lenz , G. Lamour , et al., “Continuous Self‐repair Protects Vimentin Intermediate filaments From Fragmentation,” Proceedings of the National Academy of Sciences of the United States of America 122, no. 24 (2025): 2417660122, 10.1073/pnas.2417660122. PMC12184399 40512788

[134] P. A. Coulombe , O. Bousquet , L. Ma , S. Yamada , and D. Wirtz , “The “Ins” and “Outs” of Intermediate Filament Organization,” Elsevier Current Trends 10 (2000): 420–428, 10.1016/S0962-8924(00)01828-6. 10998598

[135] E. Infante and S. Etienne‐Manneville , “Intermediate filaments: Integration of Cell Mechanical Properties During Migration,” Frontiers in Cell and Developmental Biology 10 (2022): 951816, 10.3389/FCELL.2022.951816. 35990612 PMC9389290

[136] L. M. Godsel , R. P. Hobbs , and K. J. Green , “Intermediate Filament Assembly: Dynamics to Disease,” Trends in Cell Biology 18, no. 1 (2008): 28–37, 10.1016/j.tcb.2007.11.004. 18083519

[137] A. Pora , S. Yoon , G. Dreissen , et al., “Regulation of Keratin Network Dynamics by the Mechanical Properties of the Environment in Migrating Cells,” Scientific Reports 10, no. 1 (2020): 4574–, 10.1038/s41598-020-61242-5. 32165652 PMC7067805

[138] J. M. Starger and R. D. Goldman , “Isolation and Preliminary Characterization of 10‐nm filaments From Baby Hamster Kidney (BHK‐21) Cells,” Proceedings of the National Academy of Sciences of the United States of America 74, no. 6 (1977): 2422–2426, 10.1073/pnas.74.6.2422. 329284 PMC432184

[139] C. L. Ho , J. L. Martys , A. Mikhailov , G. G. Gundersen , and R. K. H. Liem , “Novel Features of Intermediate Filament Dynamics Revealed by Green Fluorescent Protein Chimeras,” Journal of Cell Science 111, no. 13 (1998): 1767–1778, 10.1242/jcs.111.13.1767. 9625740

[140] K. L. Vikstrom , S. S. Lim , R. D. Goldman , and G. G. Borisy , “Steady state Dynamics of Intermediate Filament Networks,” The Journal of Cell Biology 118, no. 1 (1992): 121–129, 10.1083/jcb.118.1.121. 1618899 PMC2289530

[141] C. Guzmán , S. Jeney , L. Kreplak , et al., “Exploring the Mechanical Properties of Single Vimentin Intermediate filaments by Atomic Force Microscopy,” Journal of Molecular Biology 360, no. 3 (2006): 623–630, 10.1016/j.jmb.2006.05.030. 16765985

[142] A. Robert , M. J. Rossow , C. Hookway , S. A. Adam , and V. I. Gelfand , “Vimentin Filament Precursors Exchange Subunits in an ATP‐dependent Manner,” Proceedings of the National Academy of Sciences of the United States of America 112, no. 27 (2015): E3505–E3514, 10.1073/pnas.1505303112. 26109569 PMC4500282

[143] E. A. Shelden , Z. T. Colburn , and J. C. R. Jones , “Focusing Super Resolution on the cytoskeleton,” F1000Research 5 (2016), F1000 Faculty, 10.12688/F1000RESEARCH.8233.1. PMC4882751 27303635

[144] J. Maier , B. Traenkle , and U. Rothbauer , “Visualizing Epithelial–Mesenchymal Transition Using the Chromobody Technology,” Cancer Research 76, no. 19 (2016): 5592–5596, 10.1158/0008-5472.CAN-15-3419. 27634766

[145] K. N. Goldie , T. Wedig , A. K. Mitra , U. Aebi , H. Herrmann , and A. Hoenger , “Dissecting the 3‐D Structure of Vimentin Intermediate filaments by Cryo‐electron Tomography,” Journal of Structural Biology 158, no. 3 (2007): 378–385, 10.1016/J.JSB.2006.12.007. 17289402

[146] N. Rabiee and X. Lan , “Advancing Multicolor Super‐Resolution Volume Imaging: Illuminating Complex Cellular Dynamics,” JACS Au 5, no. 6 (2025): 2388–2419, 10.1021/jacsau.5c00314. 40575297 PMC12188487

